# The implications of socioeconomic factors on salivary bioscience methodological variables in a large pediatric multi-site study

**DOI:** 10.3389/fpubh.2023.1088043

**Published:** 2023-06-22

**Authors:** Hawa Mariko, Kristina A. Uban

**Affiliations:** ^1^Program in Public Health, Susan and Henry Samueli College of Health Sciences, University of California, Irvine, Irvine, CA, United States; ^2^Institute for Interdisciplinary Salivary Bioscience Research, University of California, Irvine, Irvine, CA, United States

**Keywords:** socioeconomic status, salivary bioscience, child and adolescent development, methodology, health inequities

## Abstract

**Introduction:**

Salivary bioscience has found increased utilization within pediatric research, given the non-invasive nature of self-collecting saliva for measuring biological markers. With this growth in pediatric utility, more understanding is needed of how social-contextual factors, such as socioeconomic factors or status (SES), influence salivary bioscience in large multi-site studies. Socioeconomic factors have been shown to influence non-salivary analyte levels across childhood and adolescent development. However, less is understood about relationships between these socioeconomic factors and salivary collection methodological variables (e.g., time of saliva collection from waking, time of day of saliva collection, physical activity prior to saliva collection, and caffeine intake prior to saliva collection). Variability in salivary methodological variables between participants may impact the levels of analytes measured in a salivary sample, thus serving as a potential mechanism for non-random systematic biases in analytes.

**Methods:**

Our objective is to examine relationships between socioeconomic factors and salivary bioscience methodological variables within the Adolescent Brain Cognitive Development Study© cohort of children aged 9–10 years old (*n* = 10,567 participants with saliva samples).

**Results:**

We observed significant associations between household socioeconomic factors (poverty status, education) and salivary collection methodological variables (time since waking, time of day of sampling, physical activity, and caffeine intake). Moreover, lower levels of household poverty and education were significantly associated with more sources of potential bias in salivary collection methodological variables (e.g., longer times since waking, collections later in the day, higher odds of caffeine consumption, and lower odds of physical activity). Consistent associations were not observed with neighborhood socioeconomic factors and salivary methodological variables.

**Discussion:**

Previous literature demonstrates associations between collection methodological variables and measurements of salivary analyte levels, particularly with analytes that are more sensitive to circadian rhythms, pH levels, or rigorous physical activity. Our novel findings suggest that unintended distortions in measured salivary analyte values, potentially resulting from the non-random systematic biases in salivary methodology, need to be intentionally incorporated into analyses and interpretation of results. This is particularly salient for future studies interested in examining underlying mechanisms of childhood socioeconomic health inequities in future analyses.

## 1. Introduction

Socioeconomic factors or status (SES) that drive health inequities are well established (1–3). However, a thorough understanding of SES-driven health inequities is needed within pediatric populations to elucidate early-life biological antecedents of adult health inequities. Previous studies among pediatric populations demonstrate multiple salivary biomarkers implicated in associations between the broader social environment and physiology, including neuroendocrine markers (e.g., alpha-amylase, cortisol, DHEA), metabolic markers (e.g., insulin, glucose), and immune markers (e.g., c-reactive protein, cytokines) (4–7). However, a number of these biomarkers rely on invasive sampling techniques, particularly blood draws, risking harm to participant-researcher rapport and overall willingness of communities to participate in biomedical research, particularly among pediatric populations. One approach to address this research gap in biological measures among pediatric studies is the use of salivary biosciences.

Salivary biospecimen technologies have grown in popularity over the last decade within research studies and clinical testing to non-invasively measure levels of analytes within diverse human populations (8). This utility is primarily due to its contextual practicality, allowing for sample collection outside of laboratory or clinical settings, as well as the non-invasiveness and feasibility of saliva sampling relative to more invasive techniques, such as phlebotomy (9–11). The many advantages of collecting salivary samples over other types of biospecimens in research include (1) being a low-cost option particularly for studies requiring multiple samples, (2) the ability for a participant to self-sample, and (3) adaptability to various field settings (10, 12–14). This method offers increased feasibility to measure physiological correlates of SES and related factors given the non-invasive nature, ease of collection of salivary samples, and reduced cost of sampling (10). These cost-saving benefits afford strengthening of study design such as sampling from more participants, increased number of collections within participants, or increased number of biomarkers assayed from each saliva sample. Further, salivary bioscience demonstrates great potential for diagnostic capability including pediatric endocrine dysfunction, cardiometabolic disease (15), monitoring lithium levels for psychiatric disorders (16), and diagnosing COVID-19 at home (17).

Additional methodological strengths of salivary sampling allow for the inclusion of communities that have been traditionally underrepresented in research and eases the burden of participation for families, improving adherence (13, 18). Certainly, a history of scientific injustices exists, disproportionately affecting low socioeconomic status and racially/ethnically minoritized communities, and driving historical and current-day underrepresentation in biomedical research that has often resulted in varying degrees of distrust of researchers (19–21). These historical and current injustices often occur when the cultural appropriateness of biological sample collection is not adequately considered (19, 22). Salivary collection is a tool that can minimize cultural insensitivities inherent in the collection of biological data, given its acceptance among diverse adolescent communities (23, 24). However, it is important to note that any biological collection can be precarious and warrants culturally and equity guided investigations. Some potential examples include: (a) some cultures or communities may feel averse to producing a saliva sample, particularly when observed by an experimenter, and may prefer other biological methods over saliva; (b) age of study sample matters, with children generally exhibiting aversion to blood sampling but willingness to produce saliva; and (c) certain cultures may perceive discarding unused saliva into waste as disrespectful. It is our recommendation that the community preferences for or against saliva collection be well understood before leveraging salivary biosciences.

Given these advantages, feasibility, and promising diagnostic future of salivary biosciences it is essential to first understand how the experimental design and saliva collection methodology should be standardized to ensure precision of measured analyte levels, particularly for the investigation of health inequities, and for increased application within pediatric research or clinical utility. Without this deeper methodological understanding, spurious differences in experimental design and methodological implementation of salivary biosciences may undermine the interpretability, accuracy, and utility of salivary analytes.

Several decisions in the experimental design can directly influence the methodology of salivary sample collections. For example, a design that rigorously standardizes collection of salivary samples can reduce or eliminate unintentional biases due to variations in collection methodological variables. These methodological decisions include how much time should be allowed between a participant’s waking time to their saliva collection time, the time of day the saliva sample is collected, the amount of physical activity allowed prior to sampling, if caffeine is consumed prior to sampling, or other oral considerations that can impact measured analyte levels (5, 8, 12, 25, 26). Standardized collection practices help eliminate unintended experimental noise, where non-biological factors may influence the composition or volume of whole unstimulated saliva (27). Without stringent standardized collection practices of how and when saliva samples are collected, leveraging salivary biosciences on a large scale may result in unintended methodological variations, which can impact the analyte levels measured in the collected saliva sample and thus take a detour from true biological levels, warranting caution (28).

Many adrenal steroid analytes demonstrate diurnal/circadian or seasonal rhythms, marked by patterns of varying levels over an extended period of time. For example, cortisol, a marker of psychological stress, fluctuates throughout the day, peaking approximately 30–45 min after waking followed by tapering levels in the evening (e.g., 3–12 h after waking) (11, 29). In addition, the amount of sunlight at various points of the day drives circadian rhythms (30). Waking later in the day when sunlight is different than morning light may shift circadian phases and thus alter typical patterns of analytes.

Not only is the time since waking important, the time of day when the sample is collected is also a source of experimental variation. For example, salivary dehydroepiandrosterone (DHEA) and testosterone levels are typically highest in morning samples and drop continuously throughout the day to produce lower levels in evening samples (31–34). In addition, DHEA is implicated in physiological responses to acute stress (35, 36). Thus, saliva sampled later in the day may represent different hormonal profiles compared to morning collections given fluctuating levels with circadian patterns, or greater opportunity to experience acute stressors as the day goes on. Given these considerations, minimizing variations in collection practices or pre-collection exposures are important for making accurate conclusions about the source of differences in analyte levels. Variations in methodological factors may become increasingly problematic for obtaining precise measured analyte levels in maturing adolescent populations, especially where pubertal maturation is underlying the biological systems producing the analytes of interest.

Further, methodological variables related to lifestyle such as rigorous physical activity and caffeine intake prior to salivary sample collection may introduce bias in analyte levels by altering physiological states or the integrity of the saliva sample. Rigorous (>20 min) physical activity can alter levels of DHEA or testosterone (37), particularly in saliva samples taken during early stages of pubertal maturation when hormone levels are very low (38). Salivary DHEA levels among adolescent males have been documented to increase post-exercise, yet with varying slopes according to pubertal development (35). Caffeine intake prior to saliva sampling can impact analyte levels through a few different mechanisms, including shifting the salivary pH, increasing sample acidity, and therefore impacting the performance of certain pH-sensitive assays (5, 39), or promote bacterial growth, thereby compromising the integrity of salivary fluid (40). In addition, caffeine intake may risk dehydration in the participant that would reduce salivary flow rate, and/or activation of physiological pathways that overlap with origins of the analyte of interest, such as caffeine activating the adrenergic pathway and increasing urine concentrations of metanephrine (41–43). Although these observations are in serum or urine samples, unclear evidence on correlations of serum/urine metanephrine with salivary levels as a function of caffeine intake warrants consideration of caffeine exposure in salivary collections.

Standardized collection practices can minimize differences between and within participants in these methodological variables by regulating time of day when the saliva sample is collected, prohibiting participants from consuming caffeine or performing rigorous exercise beforehand, and standardizing the duration of saliva sampling between and within participant sampling (25). Analytes closely connected to circadian patterns may be particularly sensitive to variability in sampling times, or alterations in pH levels due to caffeine consumption. The present analysis examined relationships with several salivary methodological collection variables in a large US-based, representative pediatric cohort participating in the Adolescent Brain Cognitive Development Study© (44). In the ABCD Study, detailed data was collected on methodological variables mentioned above, but was not standardized in the collection protocol allowing for our evaluation of potential non-random methodological variation relating to saliva collection and key socioeconomic factors.

Socioeconomic factors have been of central focus for understanding health inequities. Socioeconomic factors reflect access to economic or social resources and are often represented by individual or composite measures of household income level, poverty status, parental education attainment, or occupation (45). These factors have been described in the literature to influence child developmental outcomes. Low SES has been associated with poor school readiness and academic achievement, more frequent adverse experiences, structural brain differences, and altered executive functioning (46–50). Studies investigating the relationship with SES using salivary samples among children from low SES households have noted higher baseline neuroendocrine profiles and steeper neuroendocrine trajectories over time relative to children from high SES households (51, 52).

SES has been purported to operate as a function of resource availability for a study participant (53). If collected at the home, participants may have limited access to freezers to store salivary samples, mailing resources to mail collected saliva, technology, such as text messages or phone, that would facilitate reminders to collect samples at consistent timings or more accurate collection time records without the aid of digital tools (25). Possible limited availability and access to social and economic resources may influence salivary sample collection variables when participants self-schedule throughout the day when to come into the laboratory for sampling. Thus, collections performed at a laboratory or at a study site issue the question whether collection methods differ as a function of participant resource availability.

Relationships between SES and other variables important in salivary collection, namely physical activity and caffeine consumption, have been demonstrated. Positive relationships between SES and the amount of physical activity performed among adolescents have been reported, such that low SES tends to be associated with less physical activity compared to those with a high SES (3, 54, 55). However, variations in the measurement of both SES (e.g., income-to-needs ratio, household income, parental occupation, parental education) and amount of physical activity (e.g., time or duration, frequency, school-based or extracurricular) may contribute to some null findings (55). Despite overall reductions in the amount of caffeine consumption among children and adolescents since 2000, those living at 0–99% and 100–199% of the federal poverty level have consistently consumed caffeine at higher rates compared to those living at greater than 200% of the federal poverty level (56). Particularly among children ages 6–11 years old, rates of caffeine consumption in households with low or very low food security and income-to-poverty ratios below 2.0 are significantly higher compared to households with income-to-poverty ratios above 2.0 (57). Thus, child/adolescent physical activity and caffeine consumption are a possible source of methodological variation in saliva collection when not standardized in the collection design.

Given that many analyte levels fluctuate on a circadian rhythm, patterns of saliva collections earlier or later in the day among one socioeconomic context relative to others in the study sample would suggest potential non-random systematic errors in salivary analyte values due to broader social determinants. Similarly, socioeconomic-related differences in physical activity or caffeine consumption prior to salivary sampling may serve as another mechanism for non-random systematic errors in salivary analyte levels. Without disentangling these contributors, the inclusion of these salivary analyte values in analyses would bias conclusions regarding differences in biological outcomes. Thus, it remains important to capture a greater understanding of socioeconomic influences on salivary bioscience methodology before leveraging salivary data for accurate investigation of health inequities. The present analyses will inform how special considerations need to be made when leveraging salivary analyte levels from large multi-site studies in childhood, a critical period of development when inequities during early life developmental periods, “get under the skin.”

Investigations of the relationship between salivary collection methodological variables and socioeconomic factors among child populations are limited. However, with the emergence of salivary technology we are observing widespread utilization of salivary biosciences in large cohort studies. The objective of this study was to examine the association between key socioeconomic factors (e.g., poverty status, household education, neighborhood deprivation) and key salivary sample collection methodological variables (e.g., time since waking, collection time of day, and caffeine intake and physical activity within 24 h of sampling) among a diverse and large sample of US-based children aged 9–10 years old.

## 2. Materials and methods

### 2.1. Background on study sample and sample characteristics

This analysis was performed using a sample of children aged 9–10 years at enrollment participating in a 21-site study in the United States from the Adolescent Brain Cognitive Development (ABCD) Study© Release 3.0. This dataset was selected given that it is a large-scale longitudinal (e.g., annually over the course of 10 years) pediatric collection of whole saliva via passive drool for analysis of several hormonal analytes (e.g., estradiol among females only, DHEA and testosterone among males and females). Although there have been three collection timepoints to-date in this dataset (e.g., enrollment/baseline, year 1, and year 2), this current analysis focuses on baseline measures collected in 2016–2018 only. Longitudinal change was not the focus of the *a priori* aims, and any existing methodological variation observed at baseline are most likely repeated and similar in future waves of saliva collection in this cohort.

Participants reported to the study site for salivary sample collection, where one salivary sample was collected via passive drool from each participant at each annual timepoint (58). Participants and their guardian/parent did not receive prior instruction to prepare for the saliva collection during the study visit (e.g., participants were not instructed to abstain from eating, caffeine, or vigorous exercise prior to study visit). Upon arrival at the study site, a minimum of 30 min time passed between participants’ arrival and starting the saliva collection. During this time, participants were instructed to not eat or drink anything other than water (including no mints/gum), then asked to rinse their mouth out with water 10 min prior to providing the saliva sample. If participants were given a lunch break, or arrived immediately after lunch, the protocol allowed for minimum of 60 min before sampling. Thus, the majority of saliva samples occurred ~ 60 min after a large meal (38, 58). Participants and their guardian/parent arrived at the study site for collection based on when the study site and participant schedules aligned. Current guidelines for optimal utilization of salivary bioscience recommend the notation of time of recent meal, oral health or injuries, braces, or recent loss of deciduous teeth (5). However, many of these variables were not controlled or collected in the ABCD Study given considerations for reducing participant burden, and experimentally prioritize the central aims of the ABCD study including multi-modal MRI, comprehensive profiles of adolescent substance use, and mental health assessments.

When present at the study site, a research assistant (RA) documented the arrival time of the participant, presence of parent or guardian, and the time the participant reported waking. After the RA instructed the participant to passively drool into a sample collection tube, the RA then documented the timing of the salivary sample, duration of sample collection, discoloration, or visible imperfections, as well as duration from collection to placement into a −20°C to −80°C freezer. Guardians/parents were compensated for their participation in the ABCD study, with the level of compensation being varied between study sites to account for differences in cost of living (44). Salivary samples were then shipped from study sites on dry ice, confirmed for frozen state upon arrival, and assayed by an external laboratory (59).

**Figure 1:**
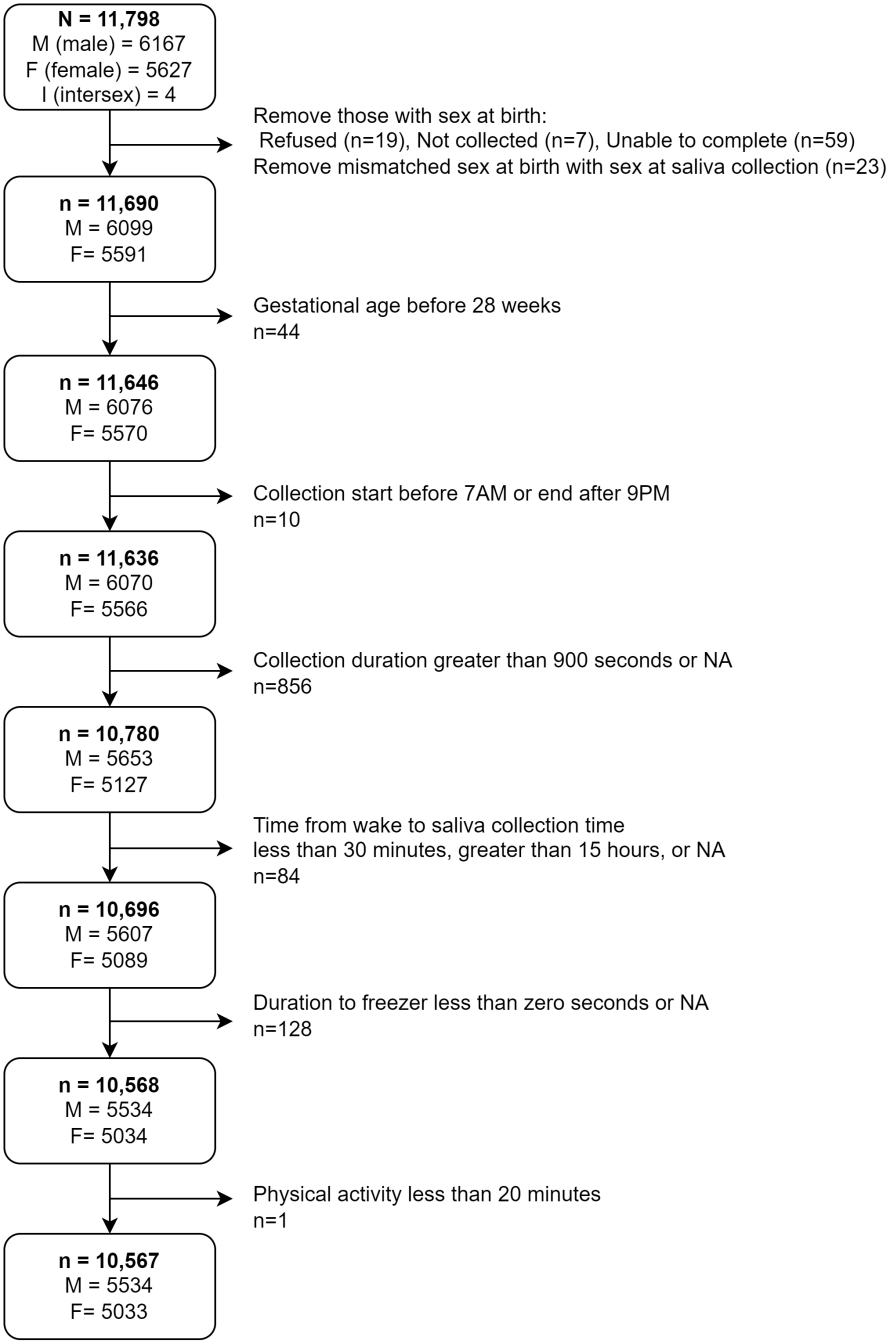
Depiction of decision tree to obtain final analytical sample. M, Male; F, Female; I, Intersex.

To reduce statistical noise within the analytic sample unrelated to sampling methodological variables, we removed participants whose biological sex at birth was not collected (*n* = 7), reported unable to complete (*n* = 59), and refused (*n* = 19) from analyses. We further cross-referenced each participant’s biological sex at birth with the biological sex reported at the time of salivary sample collection and removed those with mismatched sex (*n* = 23). We adopted this decision to cross-reference reported sex at birth with biological sex reported at Baseline collections because early ABCD protocol indicated that a participant’s sex at birth would determine which hormone panel (e.g., being inclusive or exclusive of estradiol) would be analyzed at the study visit. Only 2 participants were marked as male at birth but had missing entries at salivary sample collection. Those 2 participants were reclassified as male for analyses. We also reclassified the 4 participants reported as intersex (I) at birth (Figure 1) with the sex reported at salivary sample collection. In addition, participants with a gestational age less than 28 weeks and a reported birthweight less than 1,200 grams were removed from the analytic sample. These participants were erroneously included in the study given that the exclusion criteria required gestational age to be 28 weeks or greater. The final analytic sample consisted of *n* = 10,567, of which 5,534 were male and 5,033 were female at baseline (Figure 1).

### 2.2. Measures

#### 2.2.1. Demographic and socioeconomic variables

The inclusion of child age in statistical analyses (in months) was informed by evidence of differential sleep habits, caffeine intake, and physical activity habits between children ages 7 to 10 years old. Sleep habits including sleep duration, which may inform waking time before salivary collection, is significantly associated with child age around 9-and 10-year-olds (e.g., sleep duration decreases as child age increases) (60, 61). Further, documented significant declines in physical activity with increases in child age between ages 9 and 15 years (62–64) demonstrates a need to control for child age as a precision variable due to independent relationships with the outcome in these analyses. Regarding caffeine intake, inconsistent relationships in the literature warrant investigation in our analyses. While previous evidence demonstrates general increases in caffeine intake with increases in age, several studies (65) observed lower caffeine intake between 9- and 10-year-olds, while other studies observed similar caffeine intake among 9–10-year-olds (66). Given these existing associations, bivariate relationships were examined between child age and salivary methodological variables. After observing significant bivariate relationships (Table 1), multivariate models were adjusted for child age as a precision variable to isolate effects due to independent relationships between each predictor and the outcomes.

**Table 1:** Descriptive statistics between socioeconomic variables and salivary methodological variables.

		Time since waking (hours)		Collection time of day (hours since midnight)		Physical activity			Caffeine intake		
		Range: 0.60–14.63		Range: 7.02–20.70							
	Mean (SD)	*r_s_*	*P* ^A^	*r_s_*	*P* ^A^	<20	>20	*P* ^B^	Yes	No	*P* ^B^
						Mean (SD)	Mean (SD)		Mean (SD)	Mean (SD)	
Child age (months)	118.9 (7.5)	−0.047	<0.001	−0.041	<0.001	119 (7.5)	118.8 (7.7)	ns	119.3 (7.5)	118.9 (7.5)	ns

		Time since waking (hours)		Collection time of day (hours since midnight)		Physical activity			Caffeine intake		
Household poverty status	N	Mean (SE)	*P* ^B^	Mean (SE)	*P* ^B^	<20	>20	*P* ^C^	Yes	No	*P* ^C^
						*N* (%)	*N* (%)		*N* (%)	*N* (%)	
Deep poverty	798	5.98 (0.11)	0.01	13.05 (0.11)	<0.001	732 (7.8)	64 (5.4)	0.03	84 (12.1)	713 (7.3)	<0.001
Poverty	616	5.89 (0.13)		13.04 (0.13)		545 (5.8)	69 (5.8)		57 (8.2)	557 (5.7)	
Near poverty	1,541	5.89 (0.08)		12.99 (0.08)		1,369 (14.7)	169 (14.2)		127 (18.4)	1,413 (14.4)	
Mid income	2,358	5.84 (0.06)		12.96 (0.06)		2083 (22.3)	270 (22.8)		163 (23.6)	2,192 (22.3)	
High income	4,165	5.62 (0.05)		12.69 (0.04)		3,654 (39.1)	499 (42.0)		185 (26.7)	3,973 (40.4)	
Household education											
Less than HS	523	6.05 (0.13)	0.28	13.26 (0.13)	0.02	473 (5.1)	47 (4.0)	<0.001	59 (5.8)	461 (4.7)	<0.001
HS graduate	1,004	5.78 (0.10)		12.96 (0.10)		897 (9.6)	105 (8.8)		111 (16.0)	891 (9.1)	
Some college or associate	2,748	5.78 (0.06)		12.88 (0.06)		2,466 (26.4)	273 (23.0)		241 (34.8)	2,503 (25.5)	
College graduate	2,660	5.78 (0.06)		12.87 (0.06)		2,364 (25.3)	293 (24.7)		144 (20.8)	2,514 (25.6)	
Graduate or professional	3,596	5.76 (0.05)		12.85 (0.05)		3,118 (33.4)	469 (39.5)		137 (19.8)	3,454 (35.1)	
Household marital status											
Yes	7,082	5.87 (0.04)	0.78	12.86 (0.03)	0.05	6,216 (66.6)	848 (71.4)	0.001	392 (56.7)	6,680 (68.0)	<0.001
No	3,377	5.81 (0.05)		12.96 (0.05)		3,037 (32.6)	332 (28.0)		292 (42.2)	3,079 (31.3)	
Area deprivation index											
Quartile 1 (least deprived)	2,488	5.57 (0.06)	0.003	12.73 (0.06)	0.10	2,190 (23.5)	292 (24.6)	0.004	117 (16.9)	2,368 (24.1)	<0.001
Quartile 2	2,484	5.87 (0.06)		12.96 (0.06)		2,147 (23.0)	328 (27.6)		131 (18.9)	2,347 (23.9)	
Quartile 3	2,482	5.87 (0.06)		12.97 (0.06)		2,215 (23.7)	260 (21.9)		177 (25.6)	2,303 (23.4)	
Quartile 4 (most deprived)	2,485	5.83 (0.06)		12.87 (0.06)		2,225 (23.9)	257 (21.7)		223 (32.2)	2,258 (23.0)	

Bivariate relationships between salivary collection variables and socioeconomic variables are represented in Table 2. ^A^*P*-values of Spearman Correlation tests reflect correlations between continuous salivary collection variables and continuous child age. ^B^*P*-values of Kruskal-Wallis tests reflect associations between child age and categorical physical activity and caffeine intake. value of ps of Kruskal-Wallis tests also reflect associations between continuous salivary collection variables and categorical household poverty status, household education, household marital status, and Area Deprivation Index (ADI). ^C^*P*-values of Chi-Square test of independent reflect associations between categorical salivary collection variables and categorical socioeconomic variables. r_s_, Spearman correlation coefficient; SD, standard deviation; *P*, *p*-value; ns, non-significant.

To examine relationships between salivary collection methods with socioeconomic factors, we constructed the following measures.

Poverty status represents the household’s socioeconomic position relative to the federal poverty level (FPL). This was indexed according to the reported combined household income and the reported number of family members living from that combined income in the household. Poverty status was categorized relative to the FPL according to the following: Deep Poverty (<50%), Poverty (50– <100%), Near Poverty (100– <200%), Mid Income (200– <400%), High Income (≥400%). Although group membership across poverty status levels is imbalanced (Table 1), we made an evidence-informed decision to distinguish Deep Poverty from Poverty. From 1996 to 2011 the percentage of households living in Deep Poverty has grown 129.6% while the percentage of households in Poverty has grown 80.4% (67). Children living in Deep Poverty are at greater risk of adverse physical health and intellectual outcomes compared to children in poverty but who are not deeply poor, and children not living in poverty (68–70). Therefore, Deep Poverty is an important, unique construct of experienced poverty.

The participant’s guardian/parent self-reported their level of education, and if partnered, also reported the partner’s level of education. Household education in our analyses represents the highest level of education in the household reported by the parent. If the parent reported having a partner, then the highest level of education by either the reporting parent or the partner was included in the analyses. Otherwise, if the reporting parent did not have a partner, the single-caregiver’s reported education level was used. Previous evidence demonstrates strong positive correlations between reports of maternal education, paternal education, and the highest education level of either parent in household (71, 72). Thus, to leverage a single operationalization of household education and to reflect inclusivity in gender-neutral terminology (73), we used the highest level of education in the household reported by the parent.

Household marital status was categorized as, “yes,” if the parent reported being married. Otherwise, marital status was categorized as, “no,” if the parent reported being widowed, divorced, separated, never married, or living with partner.

Area deprivation index (ADI) was calculated as the scaled weighted sum of 17 neighborhood-level characteristics within the participant’s reported census block group. A detailed list of census variables has been summarized in Kind et al. and adapted for use in ABCD (74, 75). This includes proportion of population aged ≥25 years with <9 years of education; proportion of population aged ≥25 years with less than high school diploma; proportion of employed persons age 16+ in a “white collar” occupation; median household income; income disparity; median home value; median gross rent; median monthly mortgage; percent owner-occupied housing; percent of population age 16+ unemployed; percent of families below poverty line; percent of population below 138% of poverty line; percent of single-parent households with children <18 years; percent occupied housing units without vehicle; percent occupied units without telephone; percent occupied units without complete plumbing; percent occupied units with more than 1 person per room (74). Higher ADI scores, and thus upper quartile categorization, refer to higher levels of area deprivation, while lower quartile categorization refers to lower levels of area deprivation. Similar assessments of ADI have been widely applied in pediatric developmental research and support the validity of ADI for predicting child and family well-being (76–78). Specifically, within the ABCD cohort, many childhood outcomes such as brain structure and function, as well as body mass index, are associated with the ADI measure used in this analysis (79–81).

#### 2.2.2. Methodological variables for salivary collection

The following salivary collection variables were analyzed.

Time since waking reflects the duration of time from the participant’s self-reported time of waking to the start of the salivary sample collection documented by the RA. If a participant’s time since waking was calculated to be less than 30 min, greater than 15 h, or was missing, values were assumed to be erroneous data, and therefore were excluded from the analyses (*n* = 84). Samples with time since waking less than 30 min were removed because due to ABCD protocol, it is highly unlikely that saliva sampling occurred within this time frame. Specifically, after participants arrived at the study site, the research assistant preformed a series of pre-collection assessments, including obtaining consent/assent, explanation of saliva sampling, and conducting demographic and pubertal questionnaires before soliciting a saliva sample (82). Given that the estimated time to complete these steps was at least 30 min, samples documented to be collected within 30 min of waking are likely erroneous.

Collection time of day refers to the time of day the salivary sample collection took place at the local study site laboratory. Collections that were reported before 06:00 a.m. and after 9:00 p.m. were assumed to be erroneous data, and therefore excluded from the analyses (*n* = 10).

Physical activity was categorized dichotomously, reflecting whether the participant was vigorously physically active (sweating, breathing heavy) for at least 20 min within the 12 h prior to sampling. Participants were classified into less than 20 min of physical activity, or greater than 20 min of physical activity.

Caffeine intake was categorized dichotomously as a yes or no response, referring to whether the participant reported consuming caffeine from drink within the 12 h prior to sampling. We categorized affirmative responses coinciding with reports of non-zero milligrams of caffeine as, “yes,” and denial responses coinciding with reports of zero milligrams of caffeine as, “no,” for these analyses.

### 2.3. Statistical analyses

Associations between socioeconomic variables and salivary collection variables were examined through a series of bivariate tests. A Spearman test of correlation (*r_s_*) was performed to examine correlations between ordinally coded socioeconomic variables (Table 2). Given that neither the participant’s age in months nor the continuous salivary collection variables were normally distributed, a Spearman test of correlation (*r_s_*) was performed to examine the strength and direction of their relationship (Table 1). A Kruskal-Wallis non-parametric test of equality (*H* test statistic) was performed to identify differences in continuous salivary collection variables between levels of categorical socioeconomic variables (Table 1). A Chi-square test of independence (*X*^2^) was performed to identify associations between categorical salivary collection variables and categorical socioeconomic variables (Table 1).

**Table 2:** Bivariate correlations between socioeconomic variables.

	Household poverty status	Household education	Household marital status	ADI
Household poverty status	–	–	–	–
Household education	0.63**	–	–	–
Household marital status	0.46**	0.42**	–	–
ADI	−0.46**	−0.39**	−0.26**	–

Spearman rank correlations coefficients (r_s_) between ordinal categorical socioeconomic variables are represented below. Values range from −1 to +1 reflecting strong negative to strong positive correlations, respectively. ADI, Area Deprivation Index.

**p < 0.001.

A series of univariate and multivariate multi-level linear or logistic mixed effects models were performed to examine potential confounding effects among socioeconomic factors determining salivary collection outcomes. To account for clustering effects by study site, random intercepts were specified according to study site as level 2 and subject ID as level 1 random intercepts. Time since waking (skew = 0.5, kurtosis = −0.81) and collection time of day (skew = 0.40, kurtosis = −0.92) were log-transformed due to non-normality prior to analyses. Post-transformation skew and kurtosis for time since waking (skew = −0.30 and kurtosis = −0.76) and collection time of day (skew = 0.08, kurtosis = −1.05) were improved. Due to log transformation of continuous outcomes, beta coefficients in regression models were exponentiated to improve interpretability. The Bonferroni-corrected significance level was set to alpha = 0.0125 for 4 outcomes. Bonferroni corrected *p*-values are reported.

All tests were performed in R Statistical Software Studio version 1.3.1073 utilizing the following packages: nlme (83), car (84), piecewiseSEM (85), lubridate (86), Hmisc (87).

## 3. Results

### 3.1. Descriptive statistics

**Figure 2:**
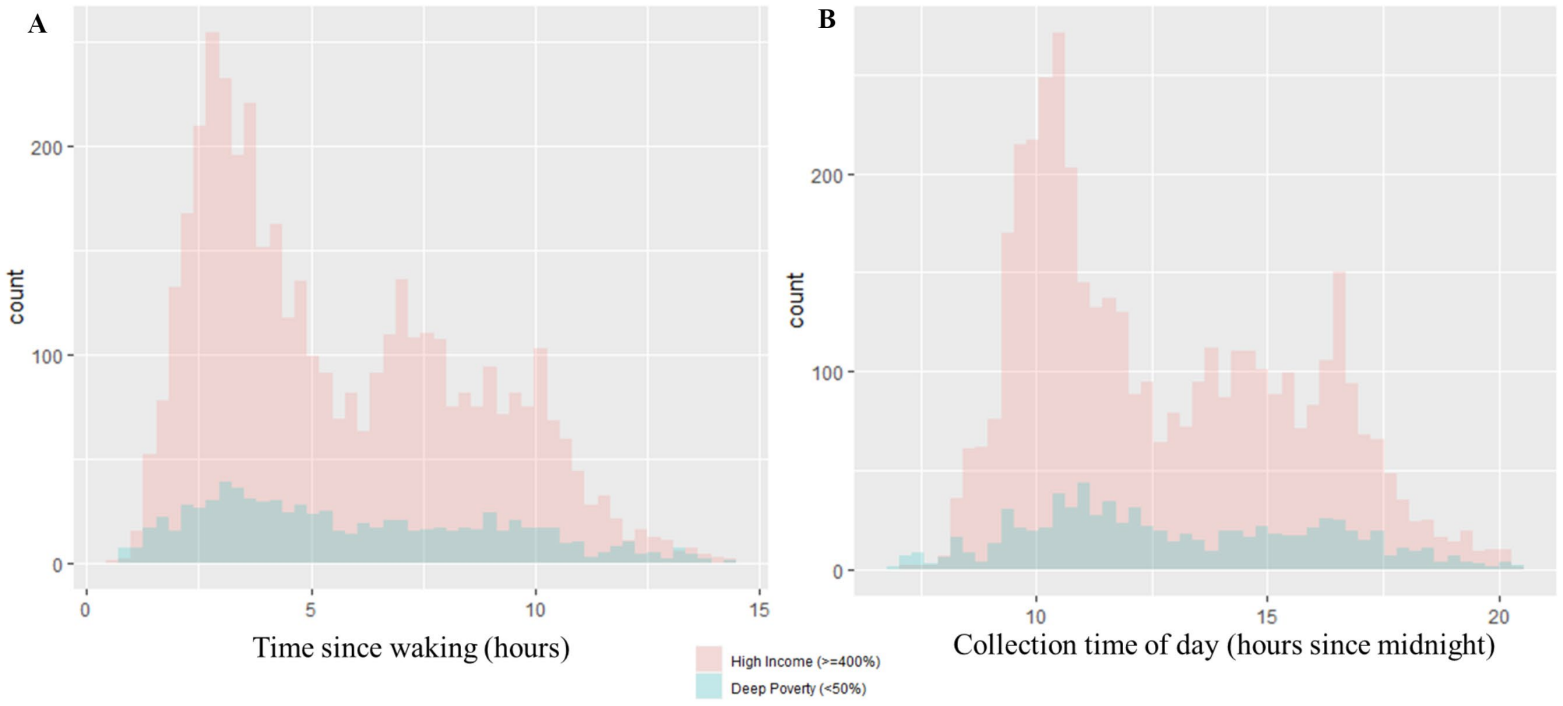
Distributions of salivary collection variables. **(A)** The distribution of time since waking in hours is represented by **(A)**. The distributions are further depicted according to poverty status (Deep Poverty versus High Income groups). Within the Deep Poverty group, the range of time since waking is 0.77–14.38 h and the median is 5.32 h. Within the High Income group, the range of time since waking is 0.60–14.33 h and the median is 4.85 h. **(B)** The distribution of collection time of day in hours since midnight is represented by **(B)**. The distributions are further depicted according to poverty status (Deep Poverty versus High Income groups). Within the Deep Poverty group, the range of collection time of day is 7.02–20.42 h and the median is 12.46 h. Within the High Income group, the range of collection time of day is 7.27–20.53 h and the median is 11.92 h.

Within the entire sample, the mean number of hours between participant waking and time of collection was 5.79 h, and the average time of collection was approximately 12 h and 53 min after midnight local time (not pictured). Time since waking and collection time of day were significantly strongly positively correlated (*r_s_* = 0.93, *p* < 0.05). No significant associations were observed between physical activity and caffeine intake (*X*^2^ = 0.25, df = 1, *p* = 0.61). A descriptive summary of salivary collection methods for the entire analytical sample, according to income group, is presented in Figure 2. Correlations between socioeconomic variables for the entire analytic sample are reflected in Table 2. All socioeconomic variables were significantly correlated with each other, albeit with ranging direction and strengths (*p*-values < 0.05, Table 2). Household poverty status was strongly positively correlated with household education (*r_s_* = 0.63, *p* < 0.05), yet moderately positively correlated with household marital status (*r_s_* = 0.46, p < 0.05). Household education was also moderately positively correlated with household marital status (*r_s_* = 0.42, p < 0.05). ADI was negatively correlated with household poverty status (*r_s_* = −0.46, p < 0.05), education (*r_s_* = −0.39, p < 0.05), and marital status (*r_s_* = −0.26, p < 0.05).

Child age (mean ± SD = 118.9 ± 7.5 months) was significantly negatively correlated with time since waking and collection time of day, albeit weakly (*p*s < 0.05, Table 1). No significant bivariate associations were observed between child age in months and physical activity nor caffeine intake.

Significant bivariate associations were observed between household poverty status and all salivary collection measures, but varying relationships between other SES factors and salivary collection measures. Mean time since waking was significantly different between levels of household poverty status (Table 1; *H* = 12.4, df = 4, *p* = 0.01), yet it was not significantly associated with household education (Table 1; *H* = 5.04, df = 4, *p* = 0.28). Household marital status was also not significantly associated with time since waking (Table 1; *H* = 0.08, df = 1, *p* = 0.78). Regarding bivariate associations at the neighborhood-level with ADI, mean time since waking (*H* = 13.9, df = 3, *p* = 0.003) was significantly different between quartiles of neighborhood deprivation (Table 1).

Additionally, while mean collection time of day was significantly different between levels of household poverty status (Table 1; *H* = 25.8, df = 4, *p* < 0.001) and household education (Table 1; *H* = 11.6, df = 4, *p* = 0.02), it was not significantly associated with household marital status (Table 1; *H* = 3.8, df = 1, *p* = 0.05) nor ADI (Table 1; *H* = 6.2, df = 3, *p* = 0.10).

Lastly, categories of physical activity and caffeine intake were not significantly independent (e.g., reject null hypothesis) of household poverty status, household education, marital status, nor ADI (Table 1). Whether or not a participant engaged in physical activity prior to sampling appeared to be significantly associated with household poverty status (*X*^2^ = 10.8, df = 4, *p* = 0.03), household education (*X*^2^ = 19.7, df = 4, *p* < 0.001), marital status (*X*^2^ = 10.3, df = 1, *p* = 0.001) and ADI (*X*^2^ = 13.3, df = 3, *p* = 0.004). In addition, caffeine consumption prior to sampling was significantly associated with household poverty status (*X*^2^ = 66.6, df = 4, *p* < 0.001), household education (*X*^2^ = 124.3, df = 4, *p* < 0.001), marital status (*X*^2^ = 35.8, df = 1, *p* < 0.001), and ADI (*X*^2^ = 45.8, df = 3, *p* < 0.001).

### 3.2. Child age

In univariate models, no significant independent relationships were observed between child age in months and time since waking and collection time of day. However, because of significant bivariate associations between child age and these salivary collection methods (Table 1), child age (months) was adjusted for in multivariate models predicting the outcomes described below.

### 3.3. Time since waking

Time since waking refers to the timeframe between the participant’s waking time and subsequent start of saliva collection. Univariate analyses demonstrated a significant 5.34% longer time since waking among deep poverty households compared to high income households (Table 3; beta = 0.05; *p* < 0.0125). ADI was not significantly associated with time since waking (Table 3).

**Table 3:** Univariate and multivariate multilevel linear models of log transformed time since waking.

	Time since waking (log)								
	Univariate			Multivariate					
				Model 1			Model 2		
	% inc or dec	Beta (SE)	*P*	% inc or dec	Beta (SE)	*P*	% inc or dec	Beta (SE)	*P*
Intercept					1.684 (0.104)	<0.001		1.684 (0.107)	<0.001
Household poverty status									
(Intercept)	374.85	1.558 (0.062)	<0.001						
Deep poverty	5.34	0.052 (0.02)	0.011	2.06	0.020 (0.020)	0.012	5.88	0.057 (0.022)	0.011
Poverty	−2.07	−0.021 (0.022)	0.352	2.27	0.022 (0.022)	0.349	−1.12	−0.011 (0.024)	0.645
Near poverty	0.36	0.004 (0.016)	0.820	1.58	0.016 (0.016)	0.823	0.60	0.006 (0.017)	0.725
Mid income	0.42	0.004 (0.013)	0.750	1.34	0.013 (0.013)	0.780	0.52	0.005 (0.014)	0.713
High income	Ref			Ref			Ref		
Area deprivation index									
Quartile 1 (least deprived)	Ref			Ref			Ref		
(Intercept)	377.10	1.563 (0.062)	<0.001						
Quartile 2	1.35	0.013 (0.015)	0.385	–	–	–	1.30	0.013 (0.016)	0.431
Quartile 3	−0.40	−0.004 (0.016)	0.796	–	–	–	−1.06	−0.011 (0.017)	0.542
Quartile 4 (most deprived)	0.55	0.005 (0.017)	0.755	–	–	–	−1.28	−0.013 (0.02)	0.530

All models adjusted for child age (months). % inc or dec refers to exponentiated beta coefficient and reflect percent increase or decrease from reference group. Due to log transformation of the outcome, beta coefficients were log transformed to improve interpretability. SE, standard error; *P*, *p*-value.

#### 3.3.1. Multivariate

When adjusting for child age or ADI in multivariate analyses, significant relationships were observed between household poverty status and a longer time since waking (Table 3; Model 1 and Model 2). Deep poverty households demonstrated a significant 2.06% longer time since waking compared to high income households, adjusting for only child age (Table 3; *β* = 0.02; *p* < 0.0125). Moreover, when adjusting for both child age and ADI, time since waking was significantly 5.88% longer among deep poverty households compared to high income households (Table 3; *β* = 0.057; *p* < 0.0125).

### 3.4. Collection time of day

Collection time of day refers to the local time of day of the salivary sample collection. In univariate analyses, deep poverty households significantly demonstrated collection start times 2.43% later in the day compared to high income households (Table 4; *β* = 0.024; *p* < 0.0125). No significant differences were observed between marital status, levels of household education, nor ADI and collection time of day in univariate analyses.

**Table 4:** Univariate and multivariate multilevel linear models of log transformed collection time of day.

	Collection time of day (log)								
	Univariate			Multivariate					
				Model 3			Model 4		
	% inc or dec	Beta (SE)	*P*	% inc or dec	Beta (SE)	*P*	% inc or dec	Beta (SE)	*P*
Intercept				1,143	2.52 (0.042)	<0.001	1,143	2.52 (0.042)	<0.001
Household marital status									
Intercept	1134.60	2.5133 (0.0259)	<0.001						
No	0.77	0.0076 (0.0043)	0.073	0.28	0.0028 (0.0052)	0.582	0.28	0.0028 (0.0053)	0.600
Yes		Ref			Ref			Ref	
Household poverty status									
(Intercept)	1129.54	2.509 (0.026)	<0.001						
Deep poverty	2.43	0.024 (0.008)	0.003	2.41	0.024 (0.01)	0.016	2.60	0.026 (0.01)	0.013
Poverty	0.02	0.0002 (0.009)	0.985	0.08	0.001 (0.01)	0.940	0.50	0.005 (0.011)	0.633
Near poverty	0.54	0.005 (0.006)	0.375	0.77	0.008 (0.007)	0.274	0.90	0.009 (0.007)	0.222
Mid income	0.66	0.007 (0.005)	0.202	0.75	0.008 (0.006)	0.173	0.89	0.009 (0.006)	0.125
High income		Ref			Ref			Ref	
Household education									
(Intercept)	1133.21	2.512 (0.026)	<0.001						
Less than HS	0.39	0.004 (0.01)	0.685	−1.07	−0.011 (0.013)	0.409	−1.42	−0.014 (0.014)	0.299
HS graduate	1.23	0.012 (0.007)	0.090	0.64	0.006 (0.009)	0.493	0.77	0.008 (0.01)	0.434
Some college or associate	0.37	0.004 (0.005)	0.465	−0.75	−0.007 (0.006)	0.236	−0.79	−0.008 (0.007)	0.229
College graduate	0.39	0.004 (0.005)	0.438	0.23	0.002 (0.005)	0.664	0.19	0.002 (0.006)	0.729
Graduate or professional		Ref			Ref			Ref	
Area deprivation index									
Quartile 1 (least deprived)		Ref			Ref			Ref	
(Intercept)	1134.21	2.513 (0.026)	<0.001						
Quartile 2	0.75	0.008 (0.006)	0.210		–	–	0.64	0.015 (0.016)	0.320
Quartile 3	0.00	0.00002 (0.006)	0.997		–	–	−0.52	−0.007 (0.018)	0.446
Quartile 4 (most deprived)	0.14	0.001 (0.007)	0.831		–	–	−0.88	−0.01 (0.021)	0.277

All models adjusted for child age (months). % inc or dec refers to exponentiated beta coefficient and reflect percent increase or decrease from reference group. Due to log transformation of the outcome, beta coefficients were exponentiated to improve interpretability. SE, standard error; *P*, *p*-value.

#### 3.4.1. Multivariate

In multivariate analyses adjusting for child age, marital status, and household education, significant relationships between household poverty status and collection time of day were maintained (Table 4; Model 3). Collection start times among deep poverty households were 2.41% significantly (marginal) later in the day compared to high income households (Table 4; *β* = 0.024; *p* = 0.016). When including ADI in multivariate analyses, marginal significant relationships between household poverty status and collection time of day were still maintained (Table 4; Model 4).

### 3.5. Physical activity

Physical activity refers to any rigorous physical activity for 20 or more minutes in the 12 h prior to providing a saliva sample. In univariate analyses, significant increases were observed in the odds of physical activity with decreasing levels of poverty. Deep poverty households demonstrated 42% lower odds of physical activity within 12 h of salivary sampling compared to high income households (Table 5; OR = 0.58, 95% CI [0.44–0.77]; *p* < 0.0125). Despite a stepwise increase in odds of physical activity with lesser impoverished households, these households were still less likely to engage in physical activity relative to high income households, albeit not significantly.

**Table 5:** Univariate and multivariate multilevel logistic models of physical activity.

	Physical activity								
	Univariate			Multivariate					
				Model 5			Model 6		
	OR	95% CI	*P*	OR	95% CI	*P*	OR	95% CI	*P*
Intercept				0.13	[0.09, 0.18]	<0.001	0.12	[0.09, 0.18]	<0.001
Household marital status									
Intercept	0.12	[0.08, 0.16]	<0.001						
No	0.78	[0.68, 0.90]	0.001	0.88	[0.75, 1.04]	0.140	0.85	[0.72, 1.01]	0.073
Yes	Ref			Ref			Ref		
Household poverty status									
(Intercept)	0.12	[0.09, 0.17]	<0.001						
Deep poverty	0.58	[0.44, 0.77]	<0.001	0.71	[0.50, 0.99]	0.046	0.76	[0.54, 1.09]	0.134
Poverty	0.82	[0.62, 1.08]	0.156	0.95	[0.69, 1.30]	0.735	0.97	[0.70, 1.36]	0.876
Near poverty	0.83	[0.69, 1.01]	0.066	0.96	[0.77, 1.20]	0.711	0.98	[0.77, 1.23]	0.848
Mid income	0.89	[0.76, 1.05]	0.175	0.98	[0.82, 1.16]	0.793	0.99	[0.83, 1.19]	0.943
High income	Ref			Ref			Ref		
Household education									
(Intercept)	0.13	[0.09, 0.17]	<0.001						
Less than HS	0.57	[0.41, 0.79]	0.001	0.66	[0.42, 1.04]	0.075	0.65	[0.40, 1.04]	0.074
HS graduate	0.74	[0.59, 0.94]	0.013	1.01	[0.75, 1.35]	0.969	1.01	[0.75, 1.38]	0.927
Some college or associate	0.73	[0.62, 0.86]	<0.001	0.79	[0.64, 0.97]	0.022	0.78	[0.63, 0.97]	0.024
College graduate	0.84	[0.72, 0.99]	0.034	0.87	[0.73, 1.03]	0.096	0.88	[0.74, 1.04]	0.131
Graduate or professional	Ref			Ref			Ref		
Area deprivation index									
Quartile 1 (least deprived)	Ref			Ref			Ref		
(Intercept)	0.11	[0.08, 0.16]	<0.001	–	–	–			
Quartile 2	1.12	[0.93, 1.35]	0.227	–	–	–	1.23	[1.00, 1.50]	0.045
Quartile 3	0.83	[0.68, 1.01]	0.060	–	–	–	0.92	[0.73, 1.15]	0.443
Quartile 4 (most deprived)	0.84	[0.67, 1.04]	0.111	–	–	–	1.04	[0.80, 1.35]	0.779

OR, odds ratio; CI, confidence interval; *P*, *p*-value.

In univariate analyses, lower levels of household education demonstrated a significantly lower odds of physical activity compared to households with Graduate/Professional educations (Table 5; *p* < 0.05). To note, univariate relationships between household education (e.g., HS graduate and College Graduate) and physical activity were not significant after Bonferroni correction. There was a pattern of increasing odds of physical activity with higher education levels. Households with a less than HS education demonstrated a 43% reduced odds of physical activity 12 h prior to salivary sampling compared to Graduate/professional households (OR = 0.57, 95% CI [0.41–0.79]; p < 0.0125). Households with a HS graduate, Some College/Associate, or College education demonstrated a respective 26, 27, and 16% reduced odds of physical activity compared to the reference group (Table 5).

ADI was not significantly associated with physical activity in univariate analyses (Table 5).

#### 3.5.1. Multivariate

In multivariate analyses adjusting for household socioeconomic factors and ADI, relationships between household poverty status and odds of physical activity became fully attenuated (Table 5; Model 5 and Model 6).

Relationships between household education and odds of physical activity became partially attenuated. Only households with Some college/Associate education demonstrated 21% lower odds of physical activity (OR = 0.79, 95% CI [0.64–0.97]; *p* < 0.05) within 12 h of salivary sampling compared to households with Graduate/Professional educations (Table 5). This result however is not significant after Bonferroni correction.

Despite univariate non-significance between ADI and physical activity, a marginally significant relationship between ADI and physical activity emerged in multivariate analyses adjusting for household marital status, household poverty status, and household education. An ADI in quartile 2 (e.g., moderately deprived neighborhood) was significantly associated with 1.23 higher odds of physical activity compared to an ADI in quartile 1 (least deprived) (OR = 1.23, 95% CI [1.00–1.50]; *p* < 0.05). These results are not significant after Bonferroni correction.

### 3.6. Caffeine intake

Caffeine intake refers to the child’s self-report of any caffeinated beverage during the 12 h prior to providing a saliva sample. In univariate analyses, significantly higher odds of caffeine intake was observed among lower levels of household poverty compared to high income households (Table 6; *p* < 0.0125). Deep poverty households had a 2.15 higher odds of caffeine intake 12 h prior to sampling compared to high income households (Table 6; OR = 2.15, 95% CI [1.62–2.85]; *p* < 0.0125). This same pattern was observed among poverty (OR = 1.95, 95%, CI [1.42–2.68]; *p* < 0.05), near poverty (OR = 1.86, 95% CI [1.46–2.36]; *p* < 0.0125), and mid income households (OR = 1.57, 95% CI [1.26–1.96]; *p* < 0.0125) (Table 6).

**Table 6:** Univariate and multivariate multilevel logistic models of caffeine Intake.

	Caffeine intake								
	Univariate			Multivariate					
				Model 7			Model 8		
	OR	95% CI	*P*	OR	95% CI	*P*	OR	95% CI	*P*
Intercept				0.04	[0.03, 0.05]	<0.001	0.04	[0.03, 0.05]	<0.001
Household marital status									
Intercept	1.10	[1.10, 1.10]	<0.001						
No	1.09	[1.09, 1.09]	<0.001	1.01	[0.83, 1.23]	0.908	1.00	[0.82, 1.23]	0.984
Yes	Ref			Ref			Ref		
Household poverty status									
(Intercept)	0.05	[0.04, 0.06]	<0.001						
Deep poverty	2.15	[1.62, 2.85]	<0.001	1.14	[0.80, 1.64]	0.466	1.15	[0.78, 1.67]	0.483
Poverty	1.95	[1.42, 2.68]	<0.001	1.05	[0.72, 1.53]	0.799	1.00	[0.67, 1.48]	0.990
Near poverty	1.86	[1.46, 2.36]	<0.001	1.20	[0.90, 1.58]	0.213	1.15	[0.86, 1.55]	0.348
Mid income	1.57	[1.26, 1.96]	<0.001	1.21	[0.95, 1.53]	0.119	1.18	[0.92, 1.51]	0.197
High income	Ref			Ref			Ref		
Household education									
(Intercept)	0.04	[0.03, 0.05]	<0.001						
Less than HS	2.88	[2.07, 4.01]	<0.001	2.49	[1.58, 3.91]	<0.001	2.21	[1.36, 3.57]	0.001
HS graduate	2.79	[2.14, 3.64]	<0.001	2.78	[1.98, 3.90]	<0.001	2.61	[1.83, 3.73]	<0.001
Some college or associate	2.33	[1.87, 2.90]	<0.001	2.11	[1.62, 2.75]	<0.001	1.94	[1.47, 2.55]	<0.001
College graduate	1.50	[1.18, 1.90]	<0.001	1.39	[1.08, 1.79]	0.010	1.40	[1.09, 1.81]	0.010
Graduate or professional	Ref			Ref			Ref		
Area deprivation index									
Quartile 1 (least deprived)	Ref			Ref			Ref		
(Intercept)	0.05	[0.04, 0.06]	<0.001	–	–	–			
Quartile 2	1.23	[0.94, 1.60]	0.129	–	–	–	0.99	[0.75, 1.31]	0.929
Quartile 3	1.59	[1.23, 2.05]	<0.001	–	–	–	1.09	[0.82, 1.45]	0.549
Quartile 4 (most deprived)	1.89	[1.45, 2.46]	<0.001	–	–	–	1.18	[0.87, 1.62]	0.293

OR, odds ratio; CI, confidence interval; *P*, *p*-value.

Lower levels of household education demonstrated a significantly higher odds of caffeine intake compared to households with Graduate/Professional educations (Table 6; *p* < 0.0125). Households with a less than HS education demonstrated a 2.88 higher odds of caffeine intake 12 h prior to salivary sampling compared to Graduate/professional households (OR = 2.88, 95% CI [2.07–4.01]; *p* < 0.0125). There was a pattern of decreasing odds of caffeine intake with higher education levels. Households with a HS graduate or Some College/Associate education demonstrated a respective 2.79, 2.33, 1.50 higher odds of caffeine intake compared to the reference group (Table 6).

ADI was only significantly associated with caffeine intake in univariate analyses (Table 6). Residing in highly deprived neighborhoods (e.g., quartile 3 and 4) was significantly associated with a 1.59–1.89 (*p* < 0.0125) higher odds of caffeine intake compared to participants residing in the least deprived neighborhoods (quartile 1).

#### 3.6.1. Multivariate

In multivariate analyses adjusting for household marital status, education, and ADI, relationships between household poverty status and odds of caffeine intake, as well as ADI and caffeine intake became fully attenuated (Model 7 and Model 8). However, significant relationships between household education and caffeine intake were maintained (Model 7 and Model 8).

## 4. Discussion

The findings from this study demonstrate significant associations between several key salivary methodological variables (time since waking, collection time of day, physical activity, and caffeine intake) with key socioeconomic factors (poverty status, household education, neighborhood deprivation). In general, lower levels of household poverty and education were significantly associated with salivary collection methodological variables (e.g., longer times since waking, collections later in the day, higher odds of caffeine consumption, and lower odds of physical activity). Furthermore, household socioeconomic context and neighborhood socioeconomic context were differentially associated with these variables. This indicates multiple sources of socioeconomic factors can independently introduce methodological biases when not fully standardized across data collection sites and individual participants. Together, present findings ultimately suggest that analyte levels measured from these samples may be impacted by non-random systematic methodological biases, particularly among analytes sensitive to variability in pH levels (e.g., caffeine in sample), physical activity/exercise, or circadian patterns. Leveraging this large salivary data set will require additional care when leveraging salivary analytes in future examination of early life antecedents of health inequities. Finally, only a subset of key socioeconomic factors and salivary sampling methodological variables were assessed in the present analyses, therefore other factors that drive health inequities may impact additional salivary methodological variables in addition to those examined in this current study.

Household poverty status was consistently significantly associated with salivary methodological variables in univariate analyses, often when comparing highly impoverished households with lesser impoverished households. These relationships were maintained in multivariate analyses when specifically predicting time since waking and collection time of day. Significant relationships between household poverty status and physical activity and caffeine intake were attenuated in multivariate analyses when adjusting for household marital status, household education, or ADI. To our knowledge, no study has examined direct relationships between household poverty status and salivary collection variables among pediatric populations. Our measure of poverty status (e.g., household income as a function of household size) may reflect more proximal measures of material or economic goods that, when scarce in impoverished households, facilitate longer durations between waking and arriving to the laboratory to provide a saliva sample, as well as sampling later in the day. With this, it may be that a reduction in economic goods associated with an impoverished household leads to unique barriers preventing an early arrival to the study site shortly after waking and earlier in the day, thereby performing salivary collections in the “tail” of diurnal rhythms when levels are low. Also, later sampling times among participants from impoverished households may have been partially or fully driven by site-specific differences in access (e.g., differences in travel time and distance). Alternatively, given the semi-flexible experimental design of the cohort study, it is possible households in poverty self-selected for a later study start time over an earlier start time in anticipation of additional barriers, such as prioritizing employment responsibilities, geographical or transportation barriers, or responsibilities of other children without funds for additional childcare. Differential preferences to come into the laboratory on a weekday versus a weekend may be another contributing source to this variability and not investigated in the present analysis. Additionally, attenuated relationships with household poverty status predicting physical activity and caffeine intake after accounting for additional socioeconomic factors, such as household education or ADI, suggest that differences in likelihood of physical activity or caffeine intake may be partially attributed to a complex interaction between several socioeconomic constructs. It is possible that individual measures of SES may be less apt to capture differences compared to composite forms of SES that include income, education, and neighborhood characteristics (53, 88). While these are only some explanations, these differences in salivary sampling methodological variables may partially, yet falsely, drive future SES-related health inequities, or null findings, in observed salivary analyte levels that are sensitive to variability in sampling methodological variables.

Household education was not significantly associated with time since waking nor collection time of day but was significantly associated with physical activity and caffeine intake in univariate and multivariate analyses. Again, to our knowledge, no study has examined direct relationships between household education and on-site salivary collection methodological variables among adolescent populations. Even with this, Krieger et al. reported weak associations between education level and physical health status however only among those living below the poverty line (89). While this study was performed among adults and examined health status, this partially supports our non-significant findings between household education and time since waking or collection time of day. In addition, relationships in our study between household education and physical activity were only significant when comparing households with Some College/Associate education to households with a Graduate/Professional education and adjusting for household poverty status. These findings are also in line with those of Krieger et al. where level of education operates on health differentially by poverty status (89). Nonetheless, this evidence may explain why household education was sparsely related to salivary collection variables. The inclusion of both household education and household poverty status in the same statistical models potentiates confounding, given evidence of strong positive correlations between one’s education level and income (88). However, we checked variance inflation factor (VIF) values for these models, and all were below 2.09, indicating that these variables were not redundant in predicting the outcomes in this study sample.

When examining neighborhood socioeconomic contexts, significant relationships were observed with ADI when predicting multivariate odds of physical activity and univariate odds of caffeine intake, whereas ADI was not significantly associated with time since waking nor collection time of day. Cerin et al. demonstrated complex relationships between environmental factors and individual-level or household-level factors (e.g., household income and education) that impact participation in physical activity (90). Differences in performing moderate to vigorous physical activity due to area-level socioeconomic factors were significantly mediated by several individual-level factors (e.g., social support from friends and self-efficacy), but not significantly mediated by infrastructure nor area-level crime (90). While ADI is a well-validated measure of neighborhood-level socioeconomic context, there are other ways to assess this construct beyond the current version (91, 92) that may miss key characteristics that are important for understanding childhood origins of health inequities. The measure of ADI used in this study is a composite of multiple forms of area economic and resource deprivation. This indicates that relationships between area-level SES and physical activity may be partially explained by individual-level factors not recorded as part of this study. While limited in the ability to inform individual-level patterns (e.g., due to ecological fallacy), this ADI measure includes factors of basic resources (e.g., plumbing, telephone) that would not be captured by income and education alone.

### 4.1. Strengths and limitations

Despite evidence for potential non-random systematic bias in salivary sampling methodological variables in the present cohort study, several strengths of the study design were observed. First, the ABCD Study© achieved coordination among 21 sites for the successful self-collection of saliva among a large pediatric cohort repeated annually. This strength adds to both the salience of the observed findings in this nationally representative pediatric study sample and further highlights the utility of salivary bioscience research on large scales and with pediatric populations. Second, the cohort sample of children was successfully recruited from the general population, rather than a convenience sample among those presenting to a clinical site, thus adding to the heterogeneity of the cohort sample, and thereby increasing the external validity of the present findings for future large-scale salivary collections. Additionally, uncovering socioecological relationships using data obtained in a non-invasive way means that salivary biosciences are well-suited to understand public health issues, particularly among children from families underrepresented in research (93). Salivary methodological variables examined in this study are often applicable to other forms of biological sample collection measuring acutely fluctuating levels (e.g., blood, urine) for analytes that vary across time of day yet correlate with salivary levels (94–98). Thus, our results may have increased generalizability beyond saliva in this study and may occur in other large biomedical research studies. Other biological methods measuring chronic levels would not be impacted by these methodological variations (e.g., hair, nails, teeth).

Nonetheless, there are several limitations to the current analyses. First, part of the exclusion criteria for the current analytical sample was a mismatch between parental report of “biological sex at birth” and the participant endorsed a binary “biological sex/gender” at the time of saliva collection from baseline (e.g., current analyses). Unfortunately, given that sex at birth determined the hormone panel for testing prior to Year 3, this protocol misrepresents associations between estradiol and variants of male sex or gender expression by not assaying saliva samples for the assumed “female” hormone. This experimental strategy potentially excludes important dynamics in gender identification throughout pubertal maturation (99) and may limit our ability to fully understand how hormones emerge across a diversity of gender identities in the current data set. In year 3, ABCD protocol solicited the participant’s endorsement of any gender identity at saliva collection however this is not part of the Release 3.0 dataset used in these analyses. Additional gender identity specific assessments were added to the study at this Year 3 timepoint as well. After Year 3, biological males at birth endorsing a male gender identity were assessed for testosterone and DHEA only, and all other possible combinations of gender identity endorsement (including neither gender) were assessed for testosterone, DHEA, and estradiol. Future analyses using the ABCD dataset for year 3 and later should leverage the gender identity data that better capture the dynamics of gender identification with salivary hormones. Second, there are many ways to capture socioeconomic status (SES), including measures of employment or unemployment status, wealth, type or status of occupation, or numeric income level (100). The variables used in this study are mostly reflective of household economic resources and household education. Previous evidence indicates that education and poverty status represent just two of many overlapping yet distinct dimensions comprising SES, rather than being entirely reflective of SES (53). Given that SES is a dynamic, multi-dimensional construct, the exclusion of other aspects of SES may only provide a partial understanding of socioecological relationships on salivary collection methodological variables.

Another limitation is the relative difference in smaller sample size among the deep poverty and poverty groups compared to the high-income group, given that larger sample sizes are more statistically powered to detect small effect sizes. Thus, imbalances in sample sizes can bias the findings of smaller effect sizes between groups, especially where the comparison group (e.g., deep poverty or poverty) is a smaller sample size relative to the reference group (e.g., high income). The deep poverty and poverty groups are likely underpowered to detect small effects and are the most at risk for null findings. Null findings between deep poverty and poverty with salivary methodological variables in the present study should be interpreted with caution. However, the deep poverty and poverty sample size were *n* = 798 and *n* = 616, respectively, which is relatively robust for pediatric biomedical research. In addition, for many of the observed findings, the effect sizes of the significant results in this analysis are relatively moderate to small. These results may not be observable within studies with smaller sample sizes, as sample sizes may be underpowered to detect small effect sizes. Without being contextualized to specific analytes of interest, the practical application of current findings is limited.

In addition, an area-level measure such as ADI is subject to an ecological fallacy because aggregate-level patterns may not actually reflect individual-level socioeconomic measures (53, 101). Although we leverage multi-level models accounting for participant clustering by study site, we observed different relationships to salivary collection methodological variables between household income/education and ADI. One potential explanation as to why ADI was not related to time-dependent salivary collection variables is that ADI may not be as proximal to household level factors, and thus would not reflect direct relationships to time since waking or collection time of day.

Another limitation of this analyses is the focus on salivary bioscience methodological variables only. The observed relationships discovered in the present analyses were not examined further in relation to specific salivary analytes that have been assayed in the samples (e.g., DHEA, testosterone, estradiol). Associations of socioeconomic-based differences in salivary collection methodological variables with salivary analyte levels were not directly tested in the present analysis. Further, our examination of baseline relationships may also limit interpretability over time, especially with longitudinal changes in SES for a participant, changes in salivary methodological variables (e.g., sampling at different time of the day or different physical activity/caffeine intake habits as participants age), and even longitudinal changes in analyte levels. Given the breadth of research questions and corresponding analytical approaches with this dataset, associations between methodological biases and analyte levels could vary across independent and longitudinal investigations. There are important considerations for whether these relationships are stable over time. We rely on existing literature that points to interference of accurate analyte measurement due to collection methodological variables (11, 29, 30, 35–37, 39, 40). Rather, this analysis encourages researchers examining health inequities to conduct a thorough examination of salivary collection methods prior to leveraging analyte levels.

While we observed significant relationships between socioeconomic and salivary sampling methodological variables, we cannot make conclusions about magnitude and directionality of relationships to specific analytes. Based on previous literature of neuroendocrine circadian patterns (30–34), we predict that these differences in salivary sampling methodological variables will become more problematic as participants continue to mature, as circadian patterns become more pronounced with maturation, and differences in exercise and caffeine intake may grow with age as a function of key socioeconomic factors. However, not all salivary analytes demonstrate a circadian rhythm or are sensitive to changes in pH of the sample, or physical activity. Thus, some specific analytes may be relatively unaffected by the observations discovered in these analyses. Researchers should evaluate whether their salivary analytes of interest reflect the observed patterns in their own analyses, and if so, intentionally address them in analyses and interpretation of salivary analyte results.

Examination of socioeconomic factors with other salivary sampling methodological variables that were collected in ABCD were out of scope for the current analyses, including cotinine levels from first and/or second-hand tobacco exposure in the children and medications that may alter salivary flow rates. However, future studies of bio-banked salivary samples could measure cotinine directly from the sample to statistically control for these confounders. There are additional salivary sampling methodological considerations that were not fully collected in this large data set, such as participant reported factors of the oral environment (e.g., blood from sores, lost teeth, injury). Research assistants used a 5-point scale to document visible alterations in the saliva sample, including presence of discoloration from food dye or blood, and food particles. A visual inspection of the salivary sample was also conducted by professional laboratory staff during the time of assaying (e.g., Salimetrics, Carlsbad, CA, United States) to note any abnormalities in the sample. Future studies would benefit from a thorough oral health questionnaire at the time of sample collection to account for salivary sample contamination. To achieve the most rigorous use of salivary analytes, all of these methodological factors should be controlled for either through upfront experimental design in future studies, expansion of questionnaires on oral health, or with careful and intentional statistical analyses to fully understand how socioeconomic factors may drive experimental noise and interfere with results. This includes maintaining strict protocols for saliva sampling regarding time since waking, time of day, sample collection duration, abstaining from caffeine, smoking, and rigorous physical activity 12 h prior to sampling.

Lastly, the examination of race/ethnicity differences was outside of the scope of this analysis; however, we encourage investigators to consider intentionally integrating upstream measures when investigating research questions pertaining to racial and ethnic minoritized groups (102–104). For example, structural racism has been identified as an important factor of adverse health among racial and ethnic minoritized groups including adolescents (105–107). Future salivary bioscience research studies must acknowledge root causes of racial/ethnic differences in health, and should be integrated in salivary bioscience research when examining race/ethnicity particularly through collaboration with experts in structural racism.

### 4.2. Conclusion

Significant associations were observed between socioeconomic factors and salivary collection methodological variables. Specifically, lower levels of household poverty and education were significantly associated with more sources of potential bias in salivary collection methodological variables (e.g., longer times since waking, collections later in the day, higher odds of caffeine consumption, and lower odds of physical activity). These novel findings serve as a thorough cautionary tale for future analyses leveraging analyte levels from these salivary samples to examine early antecedents of health inequities, as results may reflect variations in methodological variables of salivary collections (e.g., time since waking to sampling, time of day of sampling, physical activity, and caffeine intake) and not actual biological mechanisms. Entangled contributions to biological functioning from socioeconomic factors remain a potential source of non-random systematic biases. Conclusions made about biological functioning using saliva while only accounting for salivary collection methodological variables, without the consideration of socioeconomic factors, may erroneously attribute group differences to differences in biological functioning rather than the broader upstream socioeconomic environment.

These results advance salivary bioscience research by applying a health equity perspective in considering socioeconomic factors on standardizing salivary methodology. These findings highlight the importance of developing an experimental design that standardizes salivary collections, to prevent potential unintentional non-random systematic biases in saliva sampling methodology. Specifically, our results suggest that future studies ensure participants self-collect at the same time of day, for the same collection duration, and in the absence of rigorous physical activity or caffeine consumption 12 h prior to providing a sample. If stringent sample collection protocols are not feasible, we recommend that future studies collect information on potentially important salivary methodological variables (e.g., time since waking, collection time of day, physical activity, caffeine intake, oral health, medications), utilize post-hoc statistical techniques (e.g., adjustment) to cautiously disentangle effects, and target analytes that are robust to variability in salivary methodological variables. Nonetheless, salivary samples were collected effectively in participants across 21 sites, demonstrating feasibility of guided self-sampling as a non-invasive biological specimen in a large-scale pediatric study. These samples have strong potential to be leveraged in investigations of biological mechanisms across the entire sample, yet more cautiously when leveraging factors in analyses that drive health inequities.

## Data availability statement

Publicly available datasets were analyzed in this study. This data can be found: in the NIMH Data Archive (NDA), Adolescent Brain Cognitive Development (ABCD) Study (http://dx.doi.org/10.15154/1519007).

## Ethics statement

Ethical review and approval was not required for the study on human participants in accordance with the local legislation and institutional requirements. Written informed consent to participate in this study was provided by the participants’ legal guardian/next of kin.

## Author contributions

HM and KU contributed to the design of the study. HM led analyses and writing. KU contributed to analyses and writing. All authors contributed to the article and approved the submitted version.

## Funding

Support for the preparation of this manuscript was provided by the National Institute on Alcohol Abuse and Alcoholism (award no. K01AA026889 to PI: KU) including GSR supported for HM, Pre-doctoral Candidate. Data used in the preparation of this article were obtained from the Adolescent Brain Cognitive Development (ABCD) Study (https://abcdstudy.org), held in the NIMH Data Archive (NDA). This is a multisite, longitudinal study designed to recruit more than 10,000 children aged 9–10 and follow them over 10 years into early adulthood. The ABCD Study^®^ was supported by the National Institutes of Health and additional federal partners under award numbers U01DA041048, U01DA050989, Ue01DA051016, U01DA041022e, U01DA051018, U01DA051037, U01DA050987, U01DA041174, U01DA041106, U01DA041117, U01DA041028, U01DA041134, U01DA050988, U01DA051039, U01DA041156, U01DA041025, U01DA041120, U01DA051038, U01DA041148, U01DA041093, U01DA041089, U24DA041123, U24DA041147. A full list of supporters is available at https://abcdstudy.org/federal-partners.html. A listing of participating sites and a complete listing of the study investigators can be found at https://abcdstudy.org/consortium_members/. ABCD consortium investigators designed and implemented the study and/or provided data but did not necessarily participate in the analysis or writing of this report. This manuscript reflects the views of the authors and may not reflect the opinions or views of the NIH or ABCD consortium investigators. The ABCD data repository grows and changes over time. The ABCD data used in this report came from http://dx.doi.org/10.15154/1519007. DOIs can be found at https://nda.nih.gov/study.html?id=901.

## Conflict of interest

The authors declare that the research was conducted in the absence of any commercial or financial relationships that could be construed as a potential conflict of interest.

The handling editor MG declared past co-authorships with the author KU.

## Publisher’s note

All claims expressed in this article are solely those of the authors and do not necessarily represent those of their affiliated organizations, or those of the publisher, the editors and the reviewers. Any product that may be evaluated in this article, or claim that may be made by its manufacturer, is not guaranteed or endorsed by the publisher.
